# Holographic Projection Display Enlargement via Polarization-Grating Beam Steering with Fast-Response Liquid Crystal Pi-Cells

**DOI:** 10.1021/acsphotonics.6c00353

**Published:** 2026-05-07

**Authors:** Qihao Han, Tianxin Wang, Guanxiong Zhang, Waqas Kamal, Jinge Guo, Zimo Zhao, Chao He, Steve J. Elston, Stephen M. Morris

**Affiliations:** Department of Engineering Science, 6396 University of Oxford, Parks Road, Oxford, OX1 3PJ, United Kingdom

**Keywords:** Nematic liquid crystals, pi-cells, phase shifters, polarization gratings, spatial light modulator, beam steering, holographic display

## Abstract

This paper presents a holographic display architecture that integrates polarization gratings with fast-response nematic liquid crystal (LC) pi-cells to achieve fast switching and the simultaneous visual perception of multiple high-quality discrete beam-steering positions, enabling visual enlargement of holographic projected images through spatiotemporal tiling, thereby overcoming the limited projection coverage achieved using a single spatial light modulator (SLM). The system integrates voltage-controlled nematic LC pi-cell phase shifters that dynamically modulate the polarization state to achieve precise beam steering in a selected diffraction order with 80% optical transmittance and a rapid response (<3 ms). By synchronizing the voltage driving waveforms applied to the nematic LC pi-cells with the refresh rate of the SLM, the reconstructed holographic images in the replay field can be steered among multiple spatial locations in real time. Under low-frequency driving, individual holographic subframes are sequentially displayed at distinct spatial positions. At higher driving frequencies, multiple holographic projected images are visually perceived as being displayed at the same time, creating the impression of an enlarged holographic projection display area, corresponding to a 4-fold increase in the effective display area compared with a conventional holographic projection display using a single SLM at the same far-field distance. Experimental results demonstrate beam steering among four discrete positions with millisecond-scale response time, forming either a linear array (1D) or a two-by-two spatial configuration (2D). Overall, this approach provides a scalable route toward high-speed, wide-angle, and visually large-area holographic displays through the integration of polarization gratings with fast-response nematic LC devices.

## Graphical abstract

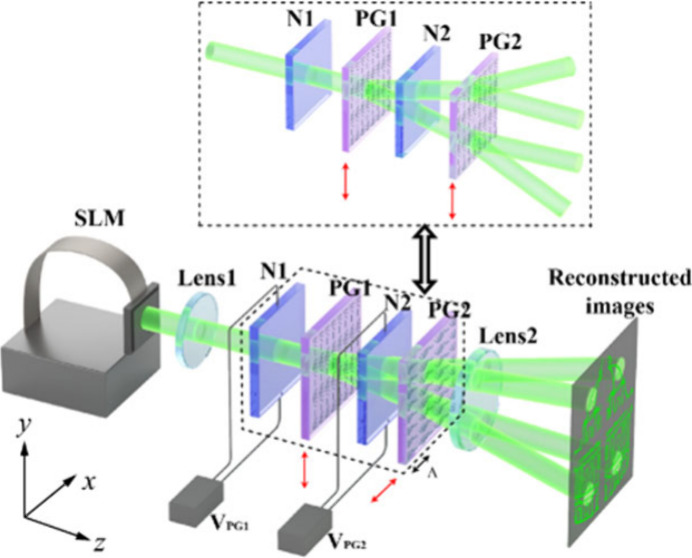

## Introduction

Holographic displays have emerged as an innovative method for producing three-dimensional (3D) visuals, providing immersive visual experiences without the need of specialized glasses or headsets.^1–3^ In contrast to conventional two-dimensional (2D) displays, which rely on pixel-based intensity modulation, holographic projection displays reconstruct full optical wavefront information on the 3D object,^4–6^ enabling accurate depth perception and parallax effects, making holographic projection displays a promising technology for applications in augmented reality (AR), virtual reality (VR),^7–10^ 3D imaging,^11,12^ and optical communications.^13^

Holographic projection systems based on SLMs are widely employed for dynamic wavefront reconstruction, in which phase or amplitude modulation is used to encode holographic information and project reconstructed optical fields into space.^14,15^ However, a key limitation of traditional holographic displays with SLMs is their narrow viewing angle (2φ), which restricts the observer’s perspective and reduces the usability of holographic content. The viewing/beam-steering angle is highly dependent on the pixel size of the SLM, as described by the equation $\varphi =\arcsin\left(\frac{\lambda }{2p_{s}}\right)$ , where λ is the optical wavelength and *p*_*s*_ is the SLM pixel pitch.^16,17^ Another significant limitation is the relatively small holographic display area (S), which is constrained by the limited active regions of common SLMs.^18^ For single-SLM systems, the 10-megapixel SLM from Holoeye with a 1.6 cm × 0.94 cm screen size, for example, offers a full viewing/beam-steering angle of 8.2°.^19^ Although larger screen sizes can increase holographic display area under identical optical conditions, they typically entail significantly higher cost.^20,21^ Expanding both the holographic projection display area in the far-field and the viewing angle is particularly important, as these improvements enlarge the viewing zone, enabling comfortable and simultaneous viewing by multiple observers.^10,22^

To address these limitations, researchers have explored both spatial and temporal multiplexing methods. In spatial multiplexing, Holobricks utilize spatial tiling of modules to achieve holographic screen enlargement with high space-bandwidth product.^6,23^ However, this method requires physically combining multiple SLM modules to form a larger aperture hologram, making it expensive and alignment-sensitive. Alternatively, temporal multiplexing approaches employ custom-made SLMs with precise control of the refresh rate and accurate synchronization that can display different holograms sequentially at high refresh rates, thereby expanding display size and improving viewing angle.^24,25^ However, brightness per view is reduced due to the temporal distribution of light energy across multiple frames. Another approach utilizes transparent holographic optical elements (HOEs) that function as diffractive or reflecting screens.^26^ This method simultaneously increases both display size and viewing angle while offering high flexibility and improved light utilization efficiency through digitally designed optical elements. Nevertheless, this approach is constrained by fabrication challenges associated with large-area, high-efficiency, multiwavelength HOEs or metasurfaces, which remain difficult and costly to manufacture with sufficient precision. Nonetheless, these limitations do not preclude the use of SLMs. Instead, they motivate the incorporation of additional optical modulation elements so that the inherent performance constraints of a single SLM can be alleviated and its capabilities more fully leveraged toward achieving large replay-field holographic projection.

Additional liquid crystal (LC)-based solutions have emerged as potential cost-effective optical elements that offer enhanced functionality of the optical modulation that, when used in combination with SLM technology, can overcome some of the intrinsic limitations as mentioned above. Liquid crystals exhibit unique electro-optical properties, characterized by their optical anisotropy and high sensitivity to external stimuli, such as electric fields and as exploited in SLM technology.^27^ By applying voltage-controlled phase modulation, beam steering of LC-based systems can also be achieved by forming LC diffraction gratings, enabling the steering of diffracted light over a wider angular range.^28,29^ Such diffraction gratings for beam steering can be realized using patterned electrodes that generate periodic electric fields.^30,31^ Previous research has successfully utilized this method to steer light for display applications, achieving enlargement of the display size.^20^ However, the relatively slow response time of such a tunable LC grating represents a significant limitation that constrains the maximum achievable display size and affects system performance. Moreover, these approaches rely on temporal scanning accumulation across different diffraction orders rather than the simultaneous perception of multiple holographic replay-field spatial positions. As a result, the slow response unavoidably reduces the quality of the holographic projection display.^20^

Additionally, LC diffraction gratings can be formed through the flexoelectric effect in nematic LC, allowing real-time control of the diffraction angle by applying varying electric fields.^29,32^ Furthermore, beam steering can be accomplished using chiral nematic LC due to their intrinsic macroscopic helical structure.^33,34^ These methods effectively steer light into different diffraction orders at various angles, offering the potential to significantly extend the viewing angle of holographic images. However, not all diffraction orders are desirable for practical applications, as higher-order diffraction often leads to energy dispersion and reduced efficiency. In many cases, it is advantageous to suppress unwanted orders and concentrate the optical power into a single diffraction order (e.g., the first order). Such single-order diffraction improves system efficiency by maximizing the usable light intensity and minimizing energy losses.

Polarization gratings (PGs) represent a class of LC device capable of optical beam-steering. These nonmechanical diffractive optical elements operate based on the Pancharatnam–Berry phase, enabling nearly 100% diffraction efficiency into a single order.^35–38^ Previous studies have utilized two PG in combination to steer light to four different locations.^39,40^ Meanwhile, the concept of combining PGs with LC optical elements has proven effective for realizing actively tunable optical devices in AR/VR display systems.^41,42^ This property can be potentially exploited in holographic projection displays to expand the effective display area and viewing zone, as will be demonstrated in the Results Section.

In this paper, we present a configuration whereby PGs are combined with electrically controllable phase shifters in the form of nematic LC pi-cells to steer holographic images generated by an SLM to different spatial locations. Fast switching is crucial for preserving synchronization with the SLM, facilitating efficient time-multiplexing and robust hologram reconstruction. By attaining high switching speeds in the nematic pi-cell LC phase shifters and accurately synchronizing the voltage drive signals with the SLM output, we achieve seamless temporal coordination of the holographic image-steering process. The concept of the proposed system is illustrated in Figure 1(a) and (b). This method effectively enlarges the holographic projection display area by combining multiple subframes generated by separate holograms under high-frequency voltage driving conditions. Furthermore, this approach is fundamentally distinct from conventional tunable-LC-grating holographic enlargement methods, which depend on patterned electrodes to form periodic structures and are therefore limited by relatively slow switching speeds. Through the integration of fast-response pi-cells and high-transmittance PGs, our system enables simultaneous visual perception of multiple beam-steering positions with fast holographic projection display switching, thereby enhancing the overall display quality.

**Figure 1:**
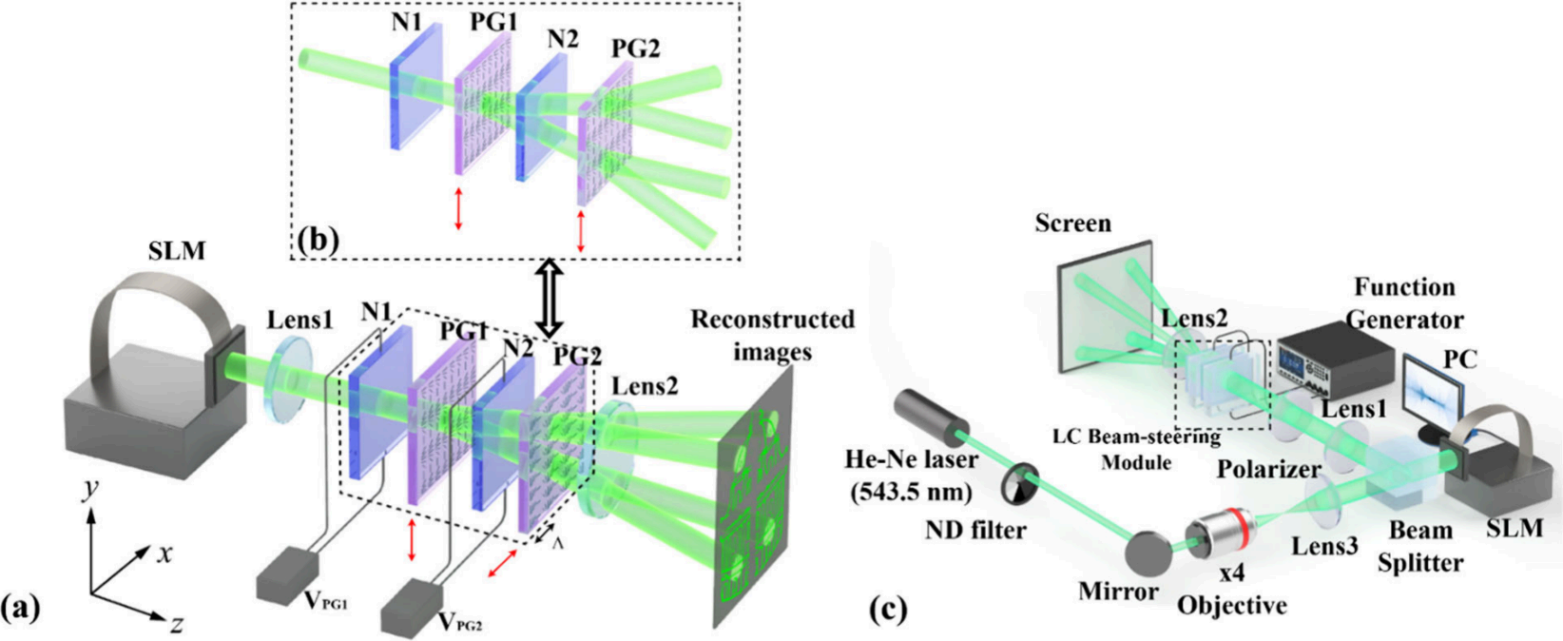
(a) Configuration for the fast switching wide-area holographic projection display. Here two polarization gratings (PGs) are arranged such that their axes are perpendicular to each other in order to generate a 2D holographic display. (b) Arrangement of the LC optical elements when the two PGs are placed such that their grating axes are aligned parallel in order to produce a one-dimensional holographic projection display. The double-headed red arrows indicate the orientations of the diffraction gratings of each PG. The *z*-axis corresponds to the optical propagation direction of the incident beam, and the PG periodicity Λ lies along the *x*-axis. (c) Schematic of the experimental arrangement for the holographic projection display system. The dashed boxes indicate the LC beam-steering module.

## Experimental Methods

### a Materials

The nematic LC cells (N1 and N2) function as optical phase shifters. They were fabricated by capillary filling the nematic LC mixture E7 (Synthon Chemical Ltd.) into pi-cells that consisted of parallel-rubbed planar alignment layers coated onto transparent conductors in the form of indium tin oxide (ITO) that were, in turn, coated onto the inner surfaces of the glass substrates (glass cells were kindly provided by Samsung). The air gap in these cells was 5 μm. A function generator was used to apply a voltage across the LC layer to tune their optical retardance, allowing the nematic pi-cells to operate as half-wave, quarter-wave, or full-wave plates, thereby controlling the polarization state of the light as it passes through the nematic pi-cell.The PGs used in this study were commercially sourced from Edmund Optics, with a design wavelength of 550 nm. Each grating, comprising the LC layer and the substrate, has a total thickness of 0.45 ± 0.04 mm, featuring a spatial frequency of 286 grooves mm^–1^. According to the manufacturer’s specifications, the first-order diffraction angle and efficiency are approximately 10° and 96%, respectively, indicating minimal leakage of the incident light into the zero-order, irrespective of its polarization. The PGs were fabricated on D263 glass substrates with an antireflective (AR) coating to minimize reflection losses.

### b Holographic Projection System

The experimental configuration for the holographic projection display is illustrated in Figure 1(c). A continuous-wave Helium–Neon (He–Ne) laser (05-LGP-193, Melles Griot) with an emission wavelength of λ = 543.5 nm was employed as a linearly polarized coherent light source. The laser provided an output power of 10 mW at this wavelength, ensuring sufficient brightness for the holographic display. To adjust the intensity as required, the beam first passed through a neutral density (ND) filter, after which it was reflected by a mirror. The beam was expanded by a ×4 objective lens in combination with Lens3 (*f*_3_ = 50 mm). Then, the incident light was directed onto the SLM (Hamamatsu Photonics, X15213 series) after passing through a beam splitter. This SLM used in this work had a resolution of 1272 × 1024 pixels, enabling high-precision phase modulation for holographic pattern generation.

The polarization state of the laser output was aligned with the operating polarization of the SLM to ensure optimal phase modulation performance. The SLM, driven by a computer, displayed the computer- generated holograms (CGH) calculated using the Gerchberg–Saxton (GS) iterative phase-retrieval algorithm. The reflected beam then passed through a polarizer to ensure linear polarization before being focused by the first lens (Lens1, *f*_1_ = 100 mm), light reflected from the SLM was then concentrated onto a set of LC optical elements consisting of two polarization gratings (PG1, PG2) and two nematic LC pi-cells (N1, N2) acting as phase shifters, controlling whether the output light is either left or right circularly polarized before it is directed onto the polarization gratings. This combination of elements, positioned between Lens1 and Lens2, enabled dynamic beam steering.

The second lens (Lens2, *f*_2_ = 25.4 mm) served to transmit and magnify the reconstructed holographic image. To further demonstrate real-time control of the holographic display via an external electric field, a flexoelectric grating can alternatively be inserted in front of the first nematic LC pi-cell. Finally, the reconstructed CGH image was projected onto a screen for observation and analysis.

## Results

To realize a large-scale holographic projection display, the first step was to dynamically steer the different CGH diffraction patterns into separate spatial locations so that when viewed together, they form a larger image. Previous studies have demonstrated that nematic LC waveplates can be used to modulate the polarization state of the incident light before it reaches the PG.^39,40^ The nematic LC cells (N1 and N2) in the configuration serve as continuously tunable phase shifters, allowing precise control over the polarization state of the light incident on each PG. Thus, each PG is paired with a nematic LC pi-cell that modulates the polarization, allowing precise steering of the CGH pattern toward the desired location.

For the PGs, when the incident light was circularly polarized, diffraction occurred exclusively into a single order (+1 or −1) with a diffraction angle of approximately 10°. The number and orientation of PGs, and the number of nematic LC phase shifters, determine the total number of beam locations projected onto the screen. When the two PGs were arranged with their grating axes aligned orthogonally, a 2 × 2 array of four distinct beam-steering locations was produced, as shown in Figure 1(a). However, when the two PGs are arranged with their grating axes aligned parallel, a linear row of four distinct beam positions was generated, as shown in Figure 1(b).

Taking the four beam spots as an example, as shown in Figure 2, the first nematic LC phase shifter (N1) operated as either a quarter-wave plate or a three-quarter-wave plate, converting linearly polarized light into either left- or right-circularly polarized light depending on the applied AC voltage amplitude in each state (*V*_PG1_S1_, *V*_PG1_S2_,*V*_PG1_S3_, *V*_PG1_S4_). The second nematic LC phase shifter (N2), on the other hand, functioned as either a half-wave or full-wave plate, reversing or maintaining the handedness of the circularly polarized light under the applied voltages of each state (*V*_PG2_S1_, *V*_PG2_,*V*_PG2_S3_, *V*_PG2_S4_). By controlling these voltages, the light can be dynamically steered into four discrete spatial locations.

**Figure 2:**
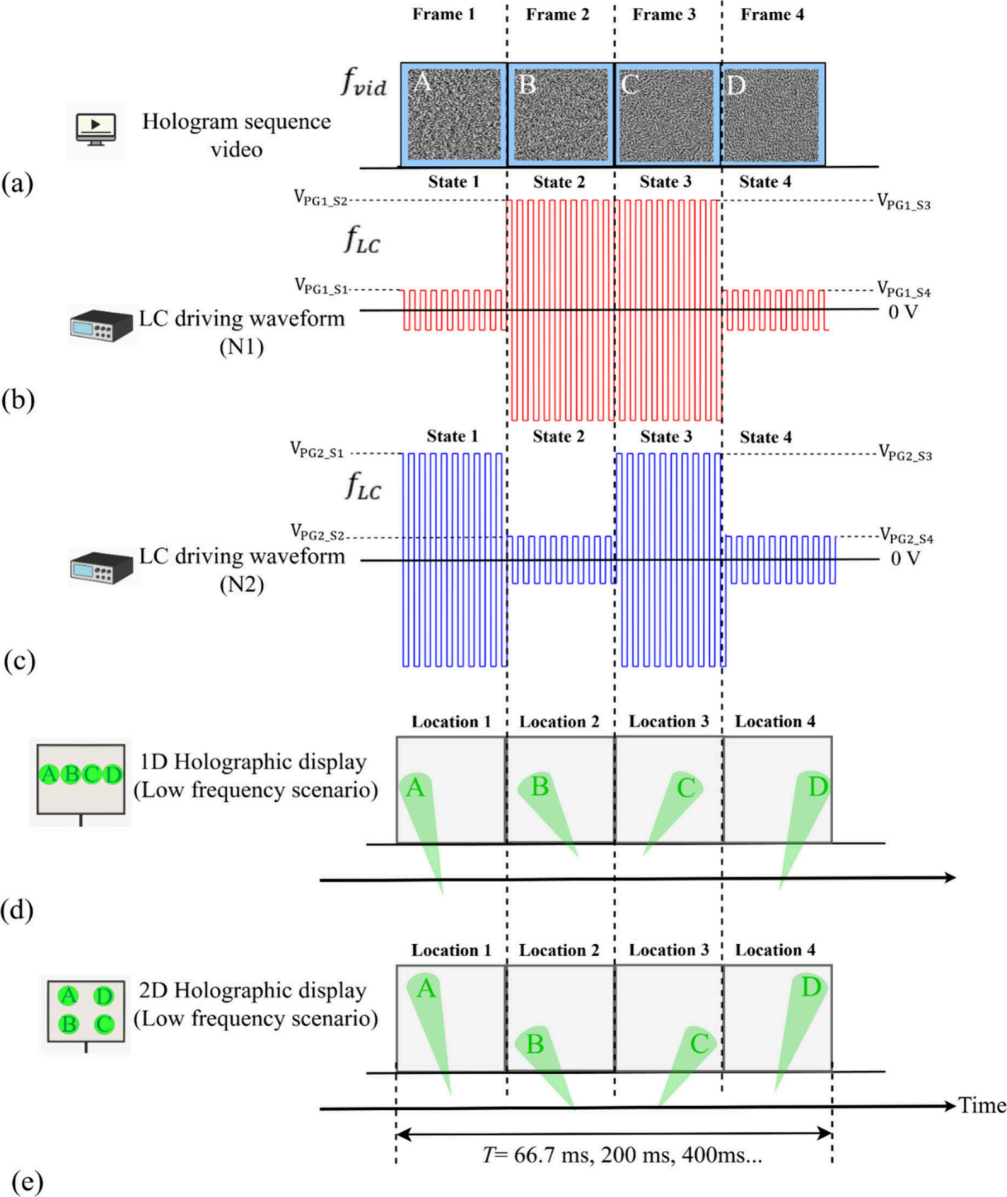
Temporal synchronization of the holographic (CGH) projection display system. (a) The hologram sequence video run on the PC consists of four subhologram frames (A–D) at a frame rate of *f*_vid_, which was mirrored on the SLM for dynamic display. (b, c) The two nematic LC pi-cells (N1 and N2) were driven by synchronized voltage waveforms that alternate between applied voltages of varying amplitudes. (d, e) Four distinct beam-steering spatial locations (A–D) of the replay field are produced through synchronized modulation between the CGH sequence and the LC driving signals, arranged in (d) a linear row (1D) and (e) a two-by-two configuration (2D).

An AC driving field was used instead of a DC field to avoid ionic screening effects, which would otherwise degrade the electro-optic performance of the LC devices. The original image can then be divided into four subimages. Each subimage is then converted into its corresponding hologram using the GS algorithm. In our work, the holograms employed are binary holograms with a black background and white foreground, as this provided a high-contrast intensity profile in which the bright regions represent strong amplitude values while the dark regions correspond to zero amplitude. Such distinct separation enhances the convergence of the iterative GS calculation and improves the accuracy of the reconstructed holographic pattern. The resulting holograms were sequentially combined to form a CGH video sequence, as shown in Figure 2(a), which was then loaded onto the SLM.

The CGH video sequence was played on a PC, while the SLM, mirroring the PC display, sequentially loaded each CGH frame. The PC and the SLM were both synchronized at a refresh rate (*f*_th_) of *f*_th_ = 60 Hz via a DVI-D interface, ensuring that the image transmission and display were synchronized. Lower frame-rate CGH sequences (e.g., *f*_*vid*_= 1, 5, 10, or 20 fps) that are integer divisors of the SLM refresh rate (*f*_th_ = 60 Hz) were automatically synchronized by frame repetition, while higher frame-rate sequences (e.g., *f*_vid_ = 120 fps) could not be displayed due to the 60 Hz refresh limit of the SLM interface. As demonstrated in Figure 2b,c, to ensure the desired beam-steering state, the driving waveform of the nematic LC pi-cells was synchronized with the CGH sequence such that each voltage state corresponded precisely to the display duration of one hologram frame. The driving frequency of the nematic LC pi-cells, denoted as *f*_LC_, was therefore set to satisfy the condition *f*_LC_ = *f*_vid_/*N*, where *N* must be an integer defining the number of voltage states per hologram cycle. The duration of each of the four states (*T*) was set to $\frac{1}{f_{\mathrm{LC}}}$ .

The temporal synchronization guaranteed that the optical phase modulation of the nematic LC pi-cells and the SLM-displayed CGH were temporally aligned, producing the expected beam-steering direction, as illustrated in Figure 2(d,e). The initial phase of the applied waveform was adjusted to enable alignment with the CGH sequence. However, slight phase misalignments were occasionally observed, primarily due to the asynchronous initialization of the CGH sequence video. Because video playback on the PC commences at an arbitrary time relative to the output cycle of the function generator, the initial phase relationship between the displayed CGH and the applied voltage waveform can vary randomly. This issue was mitigated by manually adjusting the phase of the applied waveform on the function generator until the expected beam-steering state was achieved.

Figure 2(d,e) illustrates the concept of projected holographic display of the letters A, B, C, and D, which were displayed sequentially and repeatedly at four distinct spatial locations across a single linear axis (1D) and a two-by-two spatial configuration (2D) under low frequency operation. In this case, each CGH frame corresponds to one beam steering position, causing the letters to appear one after another. When the driving frequency of the LC pi-cells was increased and synchronized with a higher video frame rate, the rapid temporal switching leads to simultaneous visual perception of the four letters across different spatial locations, thereby effectively expanding the apparent size of the holographic display. The corresponding results are presented in Figure 5(a). If a different number of beam-steering locations is desired, the number of AC voltage states must be adjusted accordingly. The replay fields were displayed on the white screen and captured using a CCD camera.

### Switching Speed Characteristics and Transmittance Analysis

The nematic pi-cells were employed as the optical phase shifters in this demonstration due to their superior switching speed compared to more conventional antiparallel rubbed nematic Fréedericksz cells. If slower LC switching were to be employed, it may lead to temporal overlap between adjacent replay fields, which can introduce ghost images in temporally multiplexed holographic displays. The fast switching of the nematic pi-cell used in this work is expected to mitigate this effect by improving the temporal separation between sequential replay fields. To demonstrate the importance of the switching speed, Figure 3(a)­(i) presents results for the Fréedericksz cell when either no voltage or only a small voltage is applied to the cell. In this case, the LC director aligns with an almost zero tilt angle (θ), corresponding to the “off” state. However, when the applied voltage exceeds the threshold for director distortion, but is not excessively high, the LC director undergoes a gradual elastic deformation across the LC layer, transitioning to the “on” state. The director reorientation during this process can be described in terms of the profile $\theta \left(z\right)=\theta_{0}\sin\frac{\pi z}{d}$ , as illustrated in Figure 3(a)­(ii), where θ­(*z*) represents the director tilt angle at position *z*, θ_0_ denotes the maximum tilt amplitude at the center of the LC layer, and *d* is the cell gap.

**Figure 3:**
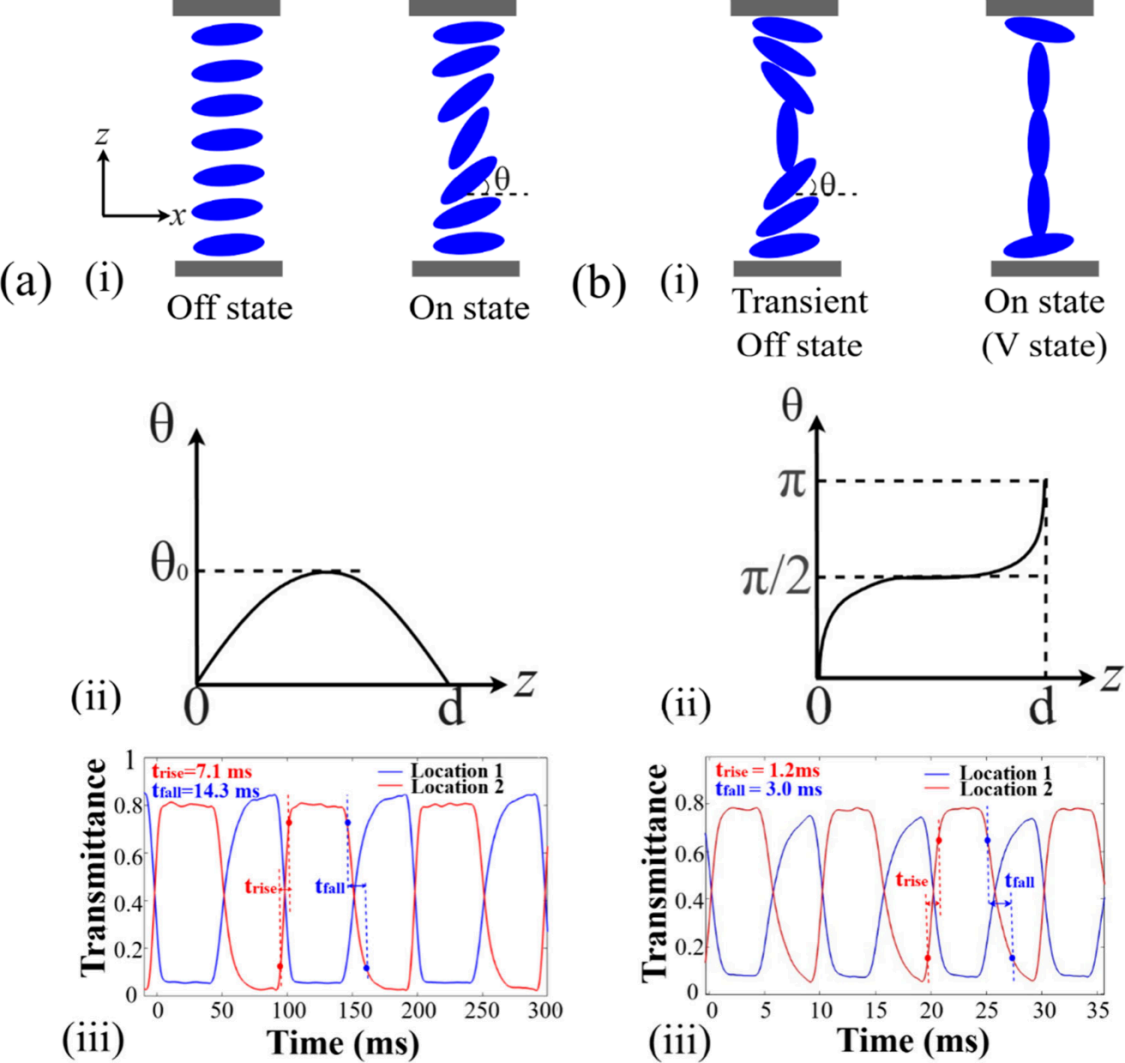
Schematic comparison of director reorientation in (a) a conventional nematic Fréedericksz (antiparallel rubbed) cell and (b) a nematic pi-cell. (i) Illustrations of the director configurations in the “off” and “on” states under an applied voltage. (ii) Corresponding director tilt angle profiles. (iii) Measured transmittance variations at location 1and location 2, illustrating the switching dynamics between two optical states. The pi-cell operated at *f*_switching_ = 100 Hz and the Fréedericksz cell operated at *f*_switching_ = 10 Hz. The measurements were performed by applying a modulated AC waveform with amplitudes alternating between 0.9 and 5 V at the specified switching frequency. All experiments were conducted at a temperature of 25 °C.

In contrast, for the pi-cell, fast-switching behavior occurs between the vertical (V) state and the transient “off” state, as shown in Figure 3(b)­(i). When a sufficiently high voltage (greater than 5 V) is applied for 1–2 s, the pi-cell enters the V state. If the voltage is momentarily reduced or removed, the LC director relaxes into a transient “off” state. In this configuration, the LC director adopts an parallel surface alignment, and the switching between these two states can be expressed as $\theta \left(z\right)=\frac{\pi z}{d}+\theta_{0}\sin\frac{2\pi z}{d}$ , as illustrated in Figure 3(b)­(ii). From these two expressions for the pi-cell and Fréedericksz, we can establish that the characteristic length scale (*D*) of the Fréedericksz cell is twice that of the pi-cell. Since the LC switching time scales as τ ∝ *D* ^2^,^27^ the pi-cell achieves a theoretically 4-fold reduction in switching time compared to the Fréedericksz cell of equal thickness.

To evaluate the switching speed of the beam-steering system, two photodiodes were used in place of the projection screen to detect light intensity at different steering locations. Location 1, corresponding to the beam-steering position where the holographic projection displays the letter “A” in Figure 2(e), and Location 2, corresponding to the position displaying the letter “B” in Figure 2(e), were selected as examples, and their corresponding photodiodes were connected to two channels of an oscilloscope. The waveform applied to N2 was disabled. Only the AC-modulated waveform corresponding to state 1 (*V*_PG1_S1_) and state 2 (*V*_PG1_S2_) of the waveform shown in Figure 2(b) was applied to the LC cell N1, enabling vertical beam steering between the two spatial positions. In this measurement, only these two stages of the full driving waveform in Figure 2(b) were selected to characterize the response speed. The applied voltage alternated between 0.9 and 5 V with a switching frequency *f*_switching_.

When the voltage applied to N1 was set to *V*_PG1_S1_ = 0.9 V, Location 1 appeared bright while Location 2 remained dark. When the voltage was increased to *V*_PG1_S2_ = 5 V, the intensity distribution reversed, with Location 1 becoming dark and Location 2 bright. The transmittance (η) at each position was defined as $\eta =\frac{I_{\mathrm{out}}}{I_{\mathrm{in}}}$ , where *I*_out_ is the detected light intensity at a given position and *I*_in_ is the incident intensity before entering the LC beam-steering module (dashed box in Figure 1). Using this arrangement, we compared our nematic pi-cell (parallel rubbed, thickness = 5 μm) with that of an antiparallel rubbed Fréedericksz cell (Samsung) of the same thickness. The results show that pi-cell exhibited a rise time of *t*_rise_ = 1.2 ms and a fall time of *t*_fall_ = 3 ms, as presented in Figure 3(b)­(iii). In contrast, the Fréedericksz cell showed slower switching characteristics, with a longer rise time of *t*_rise_ = 7.1 ms and a much longer fall time of *t*_fall_ = 14.3 ms under identical conditions, as shown in Figure 3(a)­(iii).

These results indicate that the pi-cell, when combined with a PG, enables significantly faster optical switching between two spatial locations, approximately 20 times faster than that of previously reported patterned-electrode LC beam-steering devices.^20^ However, the measured transmittance did not reach the theoretical extremes. The bright state achieved approximately 80% transmittance, while the dark state remained at 5–10%. The two primary sources of loss are residual zero-order undiffracted light leakage due to imperfections in the PG structure and the wavelength mismatch between the λ = 543.5 nm of the He–Ne laser and the design wavelength of the commercially sourced PG, reported to be approximately 4%,^40^ and Fresnel reflection losses arising from reflections at the air–glass interfaces, estimated to be approximately 4% per interface. For a configuration consisting of one PG and one nematic phase shifter separated by air, there are four air–glass interfaces. Taking these reflections into account, together with the additional 4% zero-order leakage, the estimated total transmission is approximately 81.5%. This value is close to the experimentally measured transmittance of 80%, as discussed earlier. Light scattering, nonideal retardance in the LC phase shifters, internal reflections within the LC layers, and absorption within the ITO electrodes and LC layers further contribute to transmittance loss. If the number of PG–phase shifter pairs is denoted by *M*, the total transmission can be approximately estimated as η_total_ = 0.8^*M*^. Reflection losses at the air–glass interface can be further reduced in the future by employing index-matched stacking. In addition, two approaches could be used to suppress the zero-order leakage. First, polarization gratings specifically designed for the operating wavelength could be employed. Second, careful control of the input polarization state through precise adjustment of the pi-cell drive voltages can also help suppress unwanted zero-order light.

In summary, the 80% transmittance (*M* = 1) in each state helps maintain sufficient projection intensity and contributes to the overall holographic projection image quality. Meanwhile, fast-switching pi-cells provide a significant performance advantage for the wide-angle holographic projection display system. Rapid switching is essential for maintaining synchronization with the SLM, enabling effective time-multiplexing, higher frame rates and stable hologram reconstruction. For example, if the CGH sequence operates at *f*_vid_ = 60 fps, each frame lasts 16.7 ms. In our system the pi-cells switch the steering state in about 1.3 ms, which is roughly 13 times faster than the SLM frame duration. This rapid response ensures that each CGH frame is directed to a well-defined steering state without mixing. In comparison, systems using Fréedericksz cells typically switch only about twice as fast as the SLM frame period, making them more susceptible to ghost images, blurred reconstructions, and loss of temporal synchronization. The difference becomes evident when comparing the of the holographic projection display at *f*_LC_ = 15 Hz, using the Fréedericksz cell (Supplementary Video 1) and using the pi-cell (Supplementary Video 2).

Assuming a perceptually stable frame rate of approximately 30 fps, the visual integration period is about 33 ms. With the measured switching time of the nematic LC pi-cell used in this work (1.2–3 ms, as shown in Figure 3(iii)), a theoretical maximum of approximately 11–27 replay fields could be sequentially addressed within this time window. In the present experiment, however, the system was limited by the available SLM refresh rate and the number of polarization grating modules. Nevertheless, if higher refresh-rate SLM devices and an appropriate configuration with a sufficient number of polarization gratings are employed, the proposed architecture has the potential to achieve a high level of spatiotemporal multiplexing without introducing perceptible flicker.

### Holographic Projection Display Enlargement

When the PGs were arranged with their axes aligned parallel, a linear array of four beam spots (1D) was generated, as shown in Figure 4(a). The red arrows on each PG indicate the corresponding diffraction directions. The spacing between the gratings (*L*) determines the separation (*H*) between the two central diffracted orders, given by *H* = 2*L* tan 10°. As *L* decreases, the diffracted beams move closer together and eventually overlap. Therefore, in our experiment, three diffracted orders were selected for beam steering, resulting in a total of three steerable beam positions (*N* = 3).

**Figure 4:**
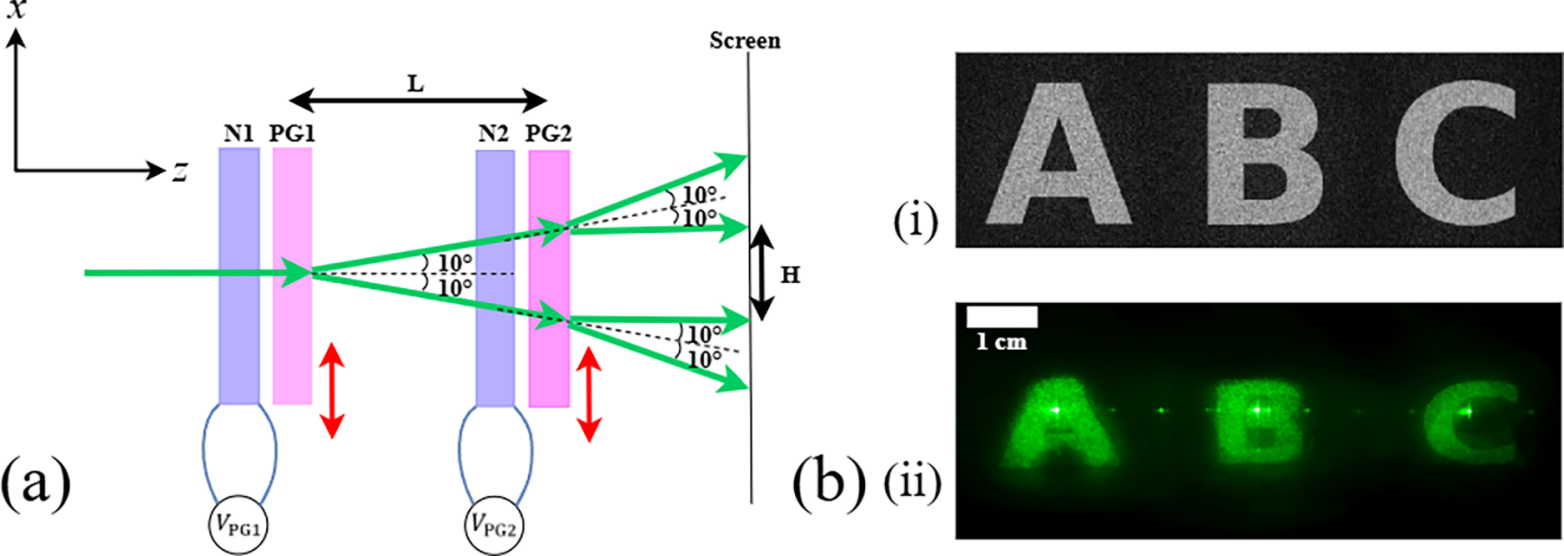
(a) Beam-steering demonstration (top view) using polarization gratings, where two parallel PGs produce four distinct beam positions. Red arrows indicate the diffraction directions of each PG, with a diffraction angle of 10°. (b) Holographic reconstruction of the letters “A–C” under three beam-steering states: (i) The target image corresponds to the loaded holographic phase, and (ii) the corresponding reconstructed holograms at different steering directions in the far-field. The camera exposure time was set to 1 s, and the distance between the screen and the LC beam-steering module was 7.5 cm.

The pi-cells were initially driven into the vertical (V) state by applying a 10 V AC field for 1–2 s. Once this stable state was established, different voltage levels in were subsequently applied to the LC phase shifters, allowing them to function as various types of wave plates to steer the diffracted light into the desired spatial locations. As shown in Table 1, When the polarization states after PG1 and PG2 are both right-circularly polarized (RCP) under applied voltages of 0.9 and 9.0 V, respectively, the image in the replay field appears at the left position. However, when the polarization states after PG1 and PG2 differ, the image shifts to the middle position. The third voltage state listed in Table 1 was not used, as it would cause the diffracted beam to overlap with that produced under the second voltage state. Finally, when the polarization states after PG1 and PG2 are both left-circularly polarized (LCP) output under voltages of 5.0 and 9.0 V, the image in the replay field moves to the right position.

**Table 1:** Three Different Voltage States Applied to the LC Phase Shifters (N1 and N2), Corresponding to Three Distinct Beam-Steering Locations on the Screen in the Far-Field^a^

voltage states	*V*_PG1_ (V)	polarization state after PG1	*V*_PG2_ (V)	polarization state after PG2	location
State 1	0.9	RCP	9.0	RCP	Location 1
State 2	0.9	RCP	2.1	LCP	Location 2
State 3 (not taken)	5	LCP	2.1	RCP	Location 3
State 4	5	LCP	9.0	LCP	Location 4

a The ‘Location’ column refers to the four beam-steering positions shown in Figure 2(d), which demonstrates the conceptual 1D holographic projection display.

By controlling the voltages applied to the LC phase shifters, the image that appeared in the replay was dynamically steered among three horizontally aligned positions. In this configuration, three voltage states were applied per hologram cycle (*N* = 3). As shown in Figure 4(b)­(ii), the holographic reconstruction of the letters “A–C” pattern was obtained. In this case, the CGH video sequence consisted of three frames, each generated from three subimages using the GS algorithm. The frame rate of the CGH video was set to *f*_vid_ = 60 fps, and the driving frequency of the waveform corresponding to the three voltage states applied to the nematic LC phase shifters was synchronized at *f*_LC_ = 20 Hz. These results demonstrate the capability of the proposed device to generate holographic projection displays arranged in a linear horizontal array.

As described previously, the holographic projection display can achieve beam steering to four distinct spatial locations (*N* = 4), forming a 2 × 2 array (2D) on the screen, when two PGs were oriented with their grating axes aligned perpendicular to one another. As shown in Table 2, by applying different voltage combinations to the LC phase shifters positioned before PG1 and PG2, the polarization states of the incident light can be precisely controlled, enabling dynamic steering of the diffracted holographic image.

**Table 2:** Four Different Voltage States Applied to the LC Phase Shifters (N1 and N2), Corresponding to Four Distinct Beam-Steering Spatial Locations in the Replay Field^a^

voltage states	*V*_PG1_ (V)	polarization state after PG1	*V*_PG2_ (V)	polarization state after PG2	location
State 1	0.9	RCP	9.0	RCP	Location 1
State 2	5.0	LCP	2.1	RCP	Location 2
State 3	5.0	LCP	9.0	LCP	Location 3
State 4	0.9	RCP	2.1	LCP	Location 4

a The ‘Location’ column refers to the four beam-steering positions shown in Figure 2(e), which demonstrates the conceptual 2D holographic projection display.

When the polarization states after PG1 and PG2 are both RCP light for applied voltages of 0.9 and 9.0 V, respectively, the reconstructed holographic pattern appears at the top-left position (Location 1). When the output after PG1 becomes LCP while that after PG2 remains RCP (5.0 and 2.1 V), the image shifts to the bottom-left (Location 2). When both PG1 and PG2 produce LCP light at voltages of 5.0 and 9.0 V, respectively, the image moves to the bottom-right (Location 3). Finally, when PG1 outputs RCP light and PG2 outputs LCP (0.9 and 2.1 V), the image in the replay field is directed to the top-right (Location 4).

As shown in Figure 5(a–c), replay field images of the letters A, B, C, and D, the University of Oxford buildings, and a panda are presented. In Figure 5(a–c)­(i), four reconstructed subimages obtained using the phase-only component of the Fourier spectrum are presented before being converted to the corresponding CGH to form the CGH video sequence. To achieve a flicker-free holographic projection display imperceptible to the human eye, the frame rate of the CGH video sequence was set to *f*_vid_ = 60 fps, and the driving frequency of the LC phase shifters was synchronized at *f*_LC_ = 15 Hz. Supporting Information S1 show the reconstructed holographic images in the far-field, where distinct gaps between adjacent subimages can be observed. By adjusting the distance between the LC beam-steering module (dashed box in Figure 1) and Lens2, the subimages can be brought closer together and seamlessly tiled, as demonstrated in Figure 5(a–c)­(ii).

**Figure 5:**
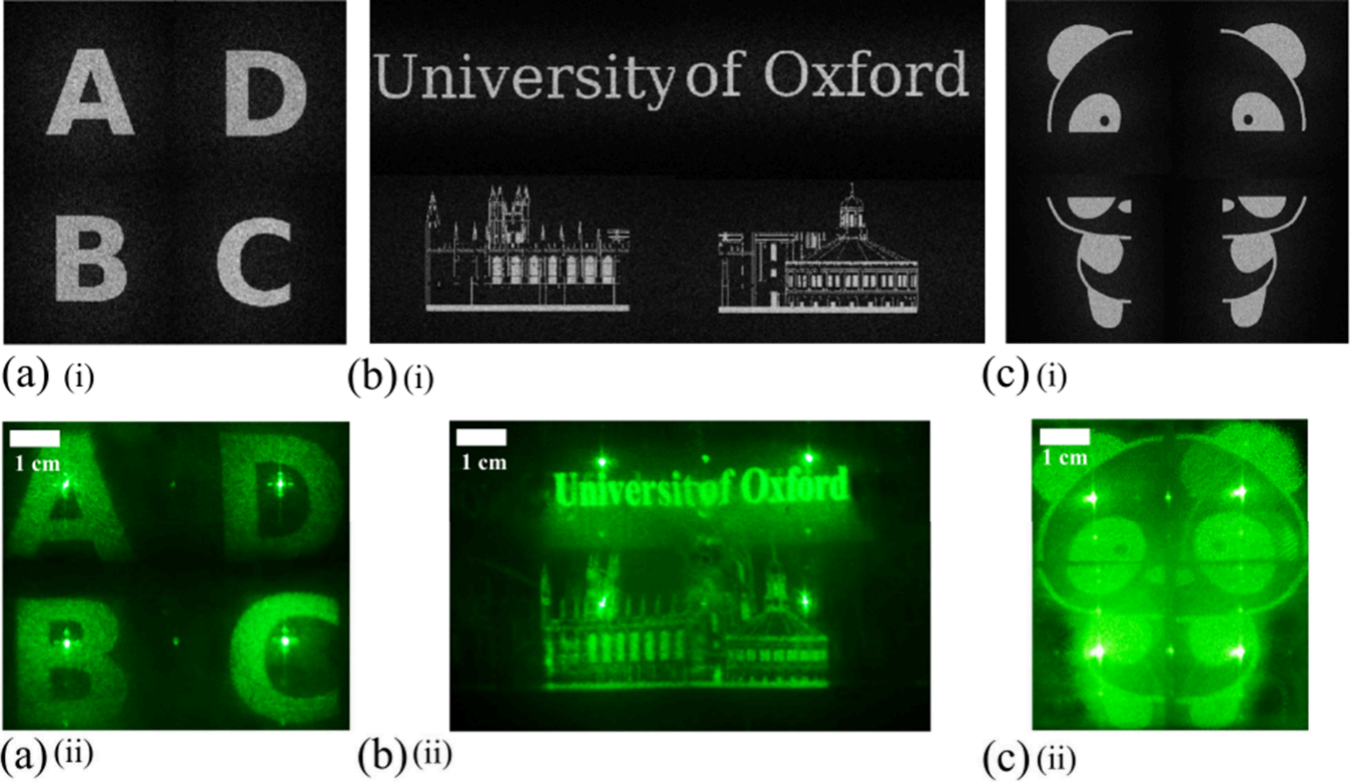
(a–c) Experimental demonstrations of holographic reconstruction of (a) letters “A–D,” (b) the University of Oxford buildings, and (c) a panda pattern, using the polarization-grating-based beam-steering system. (i) The target images correspond to the loaded holographic phase; (ii) corresponding tilted holographic reconstructions. The source image used for (b) was created with reference to the VectorStock “Oxford University Skyline” vector image,^43^ for which an Editorial License was obtained, with substantial modifications made to the final version. Permission to reuse the source image in this work was obtained through the VectorStock Editorial License. All images were recorded at a LC driving frequency of *f*_LC_ = 15 Hz. The camera exposure time was set to 1 s, and the distance between the screen and the LC beam-steering module was 7.5 cm.

Supplementary Videos 2 and 3 present the visually enlarged holographic projection of the letters A–D at LC driving frequencies of *f*_LC_ = 15 Hz and *f*_LC_ = 5 Hz, respectively. Supplementary Videos 4 and 5 present the visually enlarged holographic projection display of the panda image at LC driving frequencies of *f*_*LC*_ = 1 Hz and *f*_*LC*_ = 15 Hz, respectively. These illustrate the capability of our approach to generate enlarged holographic projections for diverse images and driving conditions. The small bright green spot observed at the center of the reconstructed images in Figure 4, Figure 5, and the originates from the zero-order leakage of the SLM, which arises due to partial refraction at its front interface.

These results successfully demonstrate the realization of holographic projection display enlargement. Previous approaches reported typically enlarge the holographic display field-of-view by employing electrically tunable LC gratings, wherein the diffraction angle is controlled by electrically modifying the LC pitch to achieve angular-domain beam steering.^20,44^ In contrast, the method proposed here operates on a fundamentally different principle. Rather than relying on continuous angular steering, our architecture achieves display enlargement through polarization-controlled spatiotemporal replay-field tiling. Specifically, fast-response pi-cells are employed to dynamically modulate the polarization state of the incident beam prior to a polarization grating, enabling distinct diffraction states to be sequentially activated and temporally multiplexed. Furthermore, polarization gratings are capable of achieving theoretically 100% diffraction efficiency into a selected diffraction order, thereby maximizing light utilization and forming an enlarged effective display region. As a result, achieving up to a 3- or 4-fold increase in display size compared with the original system without the PG–based LC beam-steering module. However, as shown in the , slight flickering remains due to the 60 fps refresh rate limitation of the SLM (*f*_th_), which prevents further increases in driving frequency and video frame rate needed to eliminate the perceptible flicker.

The brightness among the tiled subimages in each holographic reconstruction shown in Figure 5 is highly uniform. A detailed comparison between the subimages is provided in Supporting Information S2. Minor stitching artifacts may occur if the spatial separation between replay fields does not perfectly match the reconstructed tile size. However, these effects can be mitigated through appropriate system design by selecting PGs with suitable diffraction angles and applying edge apodization to smoothly blend adjacent replay fields.

As the multiplexing number *N* increases, the illumination time allocated to each replay field within one period correspondingly decreases. As a result, the perceived brightness of each replay field scales approximately inversely with the number of tiles. The enlargement in the current demonstration originates from the spatial replication of the holographic image at fixed diffraction angles generated by the polarization gratings. If PGs with different periodicities are introduced, the diffraction angles can be varied accordingly. Combining PGs with different diffraction angles would therefore provide greater flexibility for steering light to multiple spatial locations. The numerical framework and simulation results demonstrating the scalability of the PG-based beam-steering system, including the generation of 16 steering locations using PGs of different periodicities, are presented in Supporting Information S3. According to the simulation results, employing a more advanced SLM with both fast switching speeds and high refresh rates, together with the integration of additional PGs, would further visually enlarge the holographic projection display area.

## Conclusions

In this work, the enlargement of the replay field in holographic projection displays was demonstrated using a polarization grating system combined with fast-response nematic liquid crystal (LC) pi-cells. This configuration enables rapidly switchable discrete beam-steering spatial locations that are visually perceived as simultaneous, thereby extending the effective projection coverage beyond the intrinsic limitations of a single spatial light modulator. The approach presented here fundamentally differs from tunable LC grating methods for holographic projection enlargement, which rely on patterned electrodes to form periodic structures that are typically limited by slow switching speeds.

The voltage-controlled pi-cells used as phase shifters exhibit rapid switching, with a rise time of 1.2 ms, which is approximately seven times faster than a Fréedericksz (antiparallel rubbed planar-aligned) glass cell of identical thickness. Due to the characteristics of the LC diffraction grating, the optical transmittance in each state is approximately 80%, with only a small portion of power leaking into undesired diffraction orders, thereby supporting high-quality holographic projection in the replay field. By synchronizing the LC voltage driving waveforms with the computer-generated hologram sequence generated using the Gerchberg–Saxton algorithm, the reconstructed images can be dynamically directed to multiple spatial locations in real time.

Experiments confirm millisecond-scale beam steering among four discrete spatial positions, arranged either in a linear array (1D) or in a two-by-two configuration (2D), depending on the relative orientation of the polarization gratings and the polarization state imparted by the nematic LC pi-cell phase shifters. This approach enables a visual enlargement of the reconstructed holographic image by a factor of 3 to four through spatiotemporal tiling. Compared with angular tuning approaches, temporal multiplexing enables efficient replay-field stitching while maintaining high diffraction efficiency for each replay field, since polarization gratings can direct nearly all available optical power into a single diffraction order under the appropriate illumination polarization condition. However, the limited refresh rate of the SLM used in this study currently constrains further expansion of the display area. Overall, this polarization-grating-assisted system architecture provides a practical and scalable solution for realizing high-speed, wide-angle, and visually enlarged holographic projection displays. The proposed approach lays the foundation for next-generation dynamic holographic imaging systems that combine optical efficiency, temporal multiplexing, and large-aperture performance.

## Supplementary Material













## Notes

Additional experimental and simulation details, including an enlarged display with gaps between images, brightness uniformity among tiled subimages, scalability simulations of the system, and gray-to-gray switching characteristics of the SLM (PDF)
Supplementary Video 1: Dynamic 2D holographic projection display (letters A–D) using Fréedericksz liquid crystal cells, operated at a driving frequency of 15 Hz (MP4)
Supplementary Video 2: Dynamic 2D holographic projection display (letters A–D) using Pi-cells, operated at a driving frequency of 15 Hz (MP4)
Supplementary Video 3: Dynamic 2D holographic projection display (letters A–D) using Pi-cells, operated at a reduced driving frequency of 5 Hz (MP4)
Supplementary Video 4: Dynamic holographic projection display (panda image) using Pi-cells, operated at a driving frequency of 1 Hz (MP4)
Supplementary Video 5: Dynamic holographic projection display (panda image) using Pi-cells, operated at a driving frequency of 15 Hz (MP4)
