# Supplementary material for: Holographic Projection Display Enlargement via Polarization-Grating Beam Steering with Fast-Response Liquid Crystal Pi-Cells
Source: ACS Photonics. 2026 May 7;13(10):2943–53. doi: 10.1021/acsphotonics.6c00353 (PMC13195729; doi:10.1021/acsphotonics.6c00353)
Supplement: Supplementary file 1 [file ph6c00353_si_001.pdf]

Supporting Information

Holographic Projection Display Enlargement via Polarization-Grating Beam Steering with Fast-Response Liquid Crystal Pi-Cells

Qihao Han[a)], Tianxin Wang, Guanxiong Zhang, Waqas Kamal, Jinge Guo, Zimo Zhao, Chao He, Steve J. Elston and Stephen M. Morris[b)]

Department of Engineering Science, University of Oxford, Parks Road, Oxford OX1 3PJ, UK

a) Electronic mail: qihao.han@eng.ox.ac.uk;

b) Electronic mail: stephen.morris@eng.ox.ac.uk

Section S1: Enlarged display with gaps between images

Section S2. Brightness uniformity among tiled sub-images

Section S3. Simulation of the scalability of the system

Section S4. Grey-to-grey switching of SLM

Section S1. Enlarged display with gaps between images

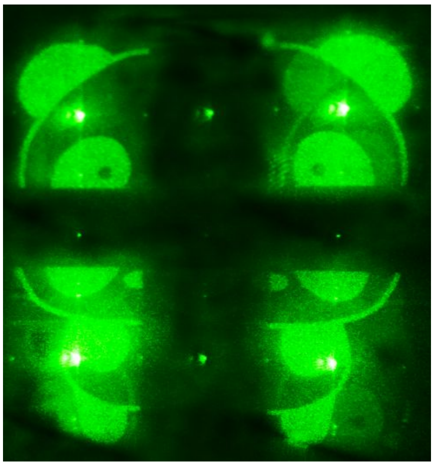

Figure S1. Experimental demonstration of holographic projection in the replay field showing distinct gaps between the separate images that make up the larger image: an image of a panda

The distance between the lens and the last polarization grating (PG2) influences the separation between individual images generated by the separate holograms. Figure S1 illustrates the reconstructed holographic projected image in the replay field for the condition where the sub-holograms are intentionally spaced apart. This configuration clearly demonstrates distinct gaps between each of the separate image elements that make up the larger image.

Section S2. Brightness uniformity among tiled sub-images

To evaluate the brightness uniformity of each sub-image, four square regions of interest (ROIs) were manually selected from the reconstructed image with the aid of MATLAB. The grayscale intensity values of the pixels within each region were then analyzed. For each ROI, the mean intensity was calculated as the average grayscale value of all pixels, representing the overall brightness of that region. The uniformity percentage was determined based on the similarity of the mean brightness values among the four selected regions. As an example, in Figure 5(c)(ii), ROIs were selected as shown in **Figure S2** from different parts of the panda's eyes in the corresponding sub-images. The selected ROIs and the calculated results are shown in **Table S2**. The analysis yields a uniformity of 97.65%, indicating that the brightness among the tiled sub-images is highly uniform.

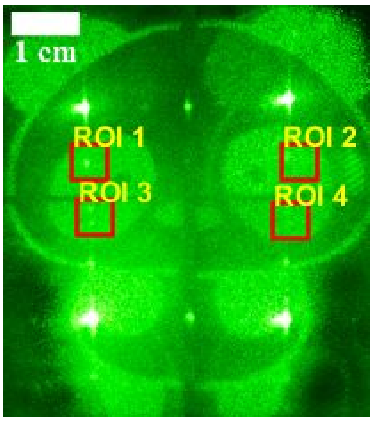

Figure S2. Selection of four regions of interest (ROIs) used for brightness uniformity analysis of the reconstructed holographic image shown in Fig. 5(c)(ii).

Table S2: Measured mean intensities of four selected regions of interest (ROIs) and the calculated brightness uniformity of the reconstructed holographic image in Fig. 5(c)(ii).

	**ROI 1**	**ROI 2**	**ROI 3**	**ROI 4**
**Means**	130.39	133.82	134.12	127.59
**Uniformity Percentage**	97.65 %			

Section S3. Simulation of the scalability of the system

The limited refresh rate of the SLM restricts the ability of the LC beam-steering module to further enlarge the size of the holographic display. To illustrate the potential scalability of the system in the future with more advanced SLM technology, simulations incorporating polarization gratings and phase shifters were performed. Here, we reconstruct the far-field intensity distribution using the phase-only component of the hologram's Fourier spectrum as the input field. The simulation was conducted following the theoretical framework described in Ref. [40]. In the model, the input beam was assumed to be either left- or right circularly polarized, corresponding to direct illumination of the PG-system without the initial phase-shifter stage.

The assembly of the optical components was modelled using two parallel-PG combinations that steer the beam along the z and y directions, respectively. Each parallel-PG combination consists of two PG aligned with their grating axes parallel to one another but with different periodicities, taking responsibility for beam deflection along one axis. As illustrated in Figure S3(a), the z-direction module, includes one PG with a geometric-phase periodicity of $\Lambda = d$, producing a diffraction angle of approximately 20°, and another PG with twice the periodicity ($\Lambda = 2d$), resulting in a diffraction angle of about 10°. In the numerical model, the latter was represented by assigning a smaller rotation rate of the optic-axis orientation, implemented by setting the total rotation angle to $\frac{\pi}{2}$ instead of π. This parallel-PG combination produces multiple diffraction orders whose distribution depends on the applied input phase and

the retardance of an intermediate LC phase shifter. An identical parallel-PG combination was used for the $y-$direction module, also comprising PGs with periodicities of $\Lambda = d$ and $\Lambda = 2d$.

The output Jones vector was sampled over a set of uniformly divided segments and propagated through the optical elements according to the following sequence,

$$E_{out} = M_2 M_{sh} M_1 E_{in},$$

where M_1 and M_2 are the Jones matrices of the two PGs, and M_{sh} represents the LC phase shifter. The output electric-field components were then Fourier-transformed to obtain the diffraction-order intensities along each direction. The diffracted intensities from the z- and y-modules were computed separately using Jones-matrix propagation and discrete Fourier transform. For each axis, four combinations of input circular polarization ($\emptyset_z$, $\emptyset_y = \pm\frac{\pi}{2}$) and LC shifter retardance ($\emptyset_{sh\_z}$, $\emptyset_{sh\_y} = \pi, 2\pi$) were applied. By combining the four states in the $z-$direction with the four states in the $y-$direction, a total of sixteen distinct diffraction outputs were generated, corresponding to sixteen spatially separated steering locations.

The simulation results are presented in Figure S3(c), where the input is the reconstructed image of the panda obtained using only the phase component of the hologram's Fourier spectrum, as shown in Figure S3(b). Because the holographic pattern is generated from a static phase-only input rather than time-sequential SLM frames, this approach demonstrates the intrinsic spatial scalability of the PG-based beam-steering system, unaffected by the refresh-rate limitation of the SLM.

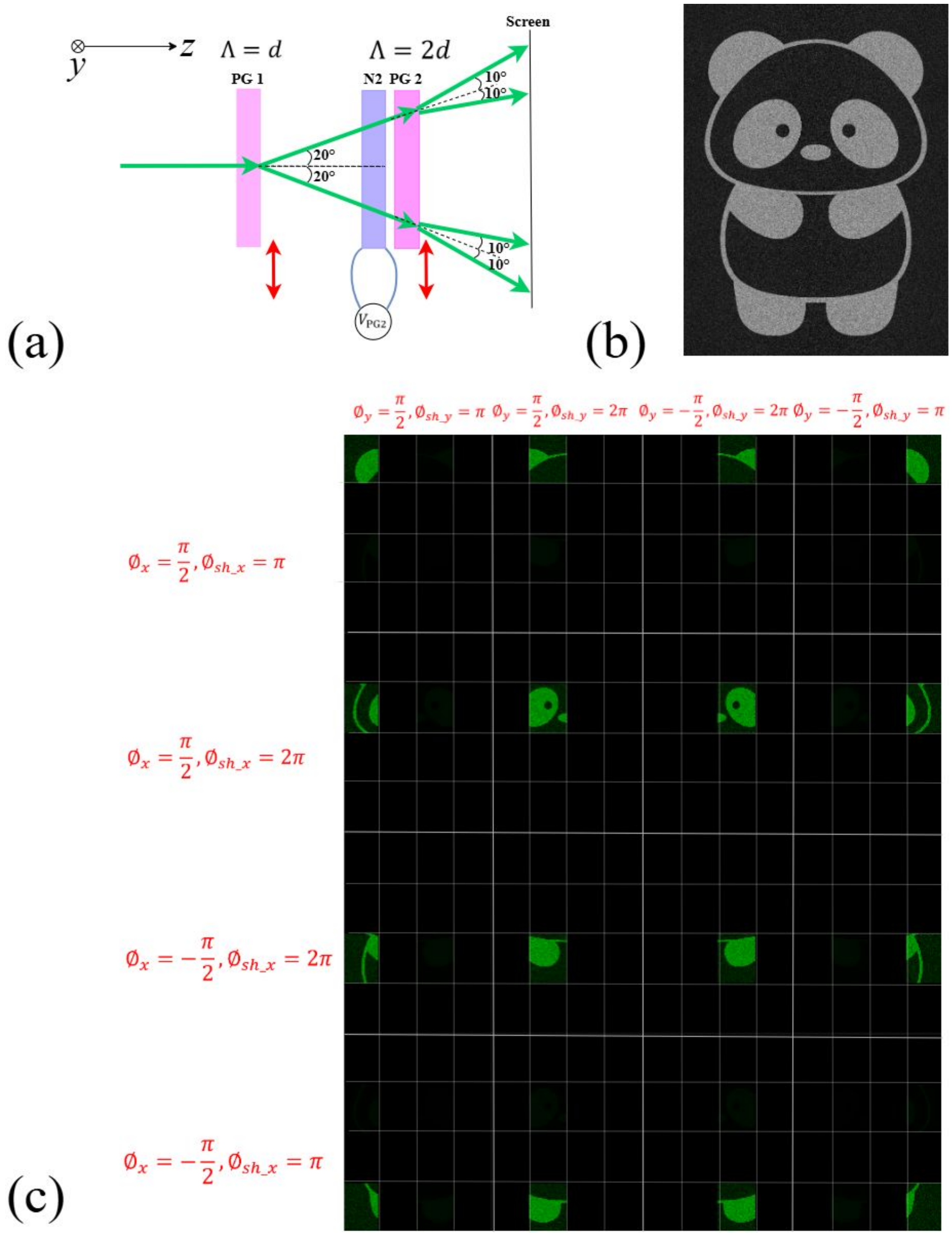

Figure S3. Demonstration of generating sixteen beam-steering positions using polarization gratings with different periodicities (Λ). (b) The target panda image corresponds to the loaded holographic phase used as the simulation input. (c) Simulated holographic projection display of the panda with sixteen separate beam steering positions.

Section S4. Grey-to-grey switching of SLM

For completeness, we have conducted additional experiments to characterize the grey-to-grey switching behavior of the Hamamatsu SLM. The input video sequences were generated to drive the SLM for grey-to-grey switching characterization. Each video consists of full-frame uniform grayscale patterns with a spatial resolution of 1272 × 1024 pixels and 8-bit grayscale depth (0–255). For each test condition, two grayscale levels were alternated periodically (e.g., 32-64, 64-128, 128-196, etc.), forming a binary temporal sequence. Each frame was held for a duration of 100 ms. The experiments were conducted at a temperature of 25 °C.

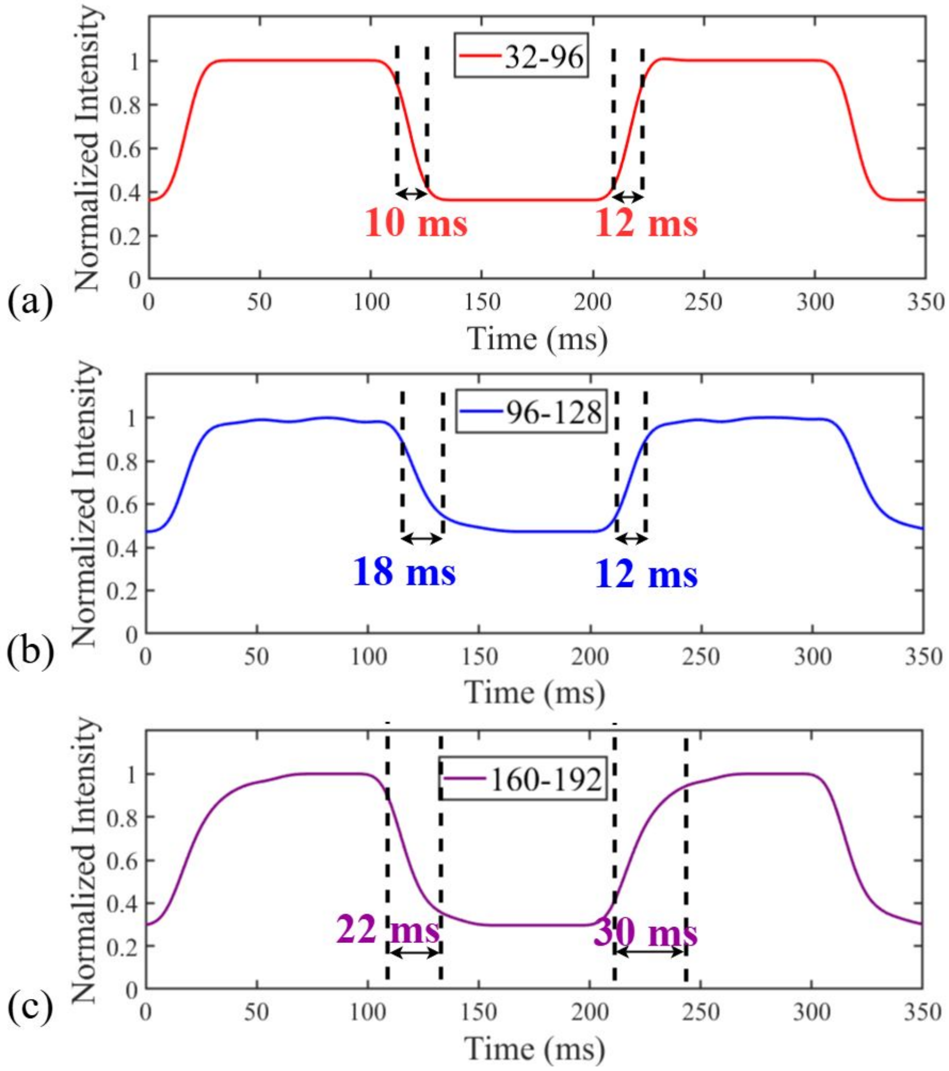

Figure S4. Measured grey-to-grey switching curves of the Hamamatsu SLM for transitions between grey levels: (a) 32- 96 (b) 96-128 (c) 160-192.

Figure S4 shows a representative grey-to-grey switching curve, while **Table S4** summarizes the measured grey-to-grey switching results.

Table S4: Measured grey-to-grey switching times of the Hamamatsu SLM. The first column denotes the initial grey levels, and the last row denotes the final grey levels. Each entry represents the switching time between the corresponding states. For example, the value of 16 ms (first row, second column) corresponds to the switching time from grey level 224 to 32.

**224**	16 ms	4 ms	8 ms	28 ms	28 ms	24 ms	N/A
**192**	12 ms	8 ms	10 ms	22 ms	22 ms	N/A	26 ms
**160**	6 ms	12 ms	14 ms	8 ms	N/A	30 ms	24 ms
**128**	12 ms	24 ms	18 ms	N/A	20 ms	30 ms	40 ms
**96**	10 ms	10 ms	N/A	12 ms	18 ms	16 ms	18 ms
**64**	8 ms	N/A	4 ms	12 ms	12 ms	10 ms	6 ms
**32**	N/A	16 ms	12 ms	22 ms	16 ms	42 ms	44 ms
	**32**	**64**	**96**	**128**	**160**	**192**	**224**
